# Clinical Comparison of a Novel Balloon-Expandable Versus a Self-Expanding Transcatheter Heart Valve for the Treatment of Patients with Severe Aortic Valve Stenosis: The EVAL Registry

**DOI:** 10.3390/jcm11040959

**Published:** 2022-02-12

**Authors:** Monica Barki, Alfonso Ielasi, Andrea Buono, Gabriele Maliandi, Mariano Pellicano, Marta Bande, Francesco Casilli, Francesca Messina, Giuseppe Uccello, Daniele Briguglia, Massimo Medda, Maurizio Tespili, Francesco Donatelli

**Affiliations:** 1 Clinical and Interventional Unit, Istituto Clinico Sant’Ambrogio, 20149 Milan, Italy; barkimonica16@gmail.com (M.B.); andrebuo@hotmail.com (A.B.); gabrielemaliandi@gmail.com (G.M.); marianopellicano@libero.it (M.P.); bande.marta@gmail.com (M.B.); francesco.casilli@grupposandonato.it (F.C.); francesca.messina@outlook.it (F.M.); giuseppe.uccello@yahoo.it (G.U.); daniele_briguglia@yahoo.it (D.B.); massimomedda2002@yahoo.it (M.M.); tespili@katamail.com (M.T.); 2 Cardiac Surgery, Department of Cardiothoracic Center, Istituto Clinico Sant’Ambrogio, University of Milan, 20122 Milan, Italy; francesco.donatelli@unimi.it

**Keywords:** transcatheter aortic valve replacement (TAVR), self-expanding CoreValve Evolut R (SE ER), balloon-expandable Myval (BE Myval), paravalvular leak (PVL), permanent pacemaker implantation (PPI)

## Abstract

Background: Transcatheter aortic valve replacement (TAVR) is an effective treatment option for patients with severe, symptomatic AS, regardless of the transcatheter heart valve (THV) implanted. Prior studies demonstrated a higher device success with lower paravalvular leak (PVL) using the balloon-expandable (BE) Sapien/XT THV vs. a self-expanding (SE) THV. However, few data are available on the performance of a novel BE THV. Purpose: to compare early clinical performance and safety of the newly available BE Myval THV (Myval, Meril Life Sciences Pvt. Ltd., India) vs. the commonly used SE (Evolut R, Medtronic) THV. Methods: A single-center, retrospective cohort analysis was performed with 166 consecutive patients undergoing TAVR from March 2019 to March 2021 for severe symptomatic AS treated with either the novel BE Myval or the SE Evolut R (ER) bioprosthesis. The primary endpoint was device success at day 30 according to the Valve Academic Research Consortium-3 (VARC-3). Secondary endpoints included 30-day all-cause mortality, cardiovascular mortality, more than mild PVL, permanent pacemaker implantation (PPI) rates and a composite of all-cause mortality and disabling stroke at 6 months. Results: Among the 166 included patients, 108 patients received the SE ER THV and 58 patients were treated with the BE Myval THV. At baseline, the two groups showed comparable demographic characteristics. The primary composite endpoint of early device success occurred in 55 patients (94.8%) in the BE Myval group and in 90 patients (83.3%) in the SE ER group (OR 3.667, 95% CI 1.094–12.14; *p* = 0.048). At day 30, the BE Myval THV group exhibited a significantly lower incidence of more than mild PVL (BE Myval 3.45% vs. SE ER 14.8%, OR 0.2, 95% CI 0.05–0.8; *p* = 0.0338), along with a lower rate of PPI (BE Myval 11% vs. SE ER 24.2%, OR 0.38, 95% CI 0.15–0.99; *p* = 0.0535). At the 6-month follow-up, the incidence of all-cause mortality and disabling stroke did not significantly differ between the two groups, while the incidence of PPI (BE Myval 11% vs. SE ER 27.5%, OR 0.32, CI 95% 0.1273–0.8; *p* = 0.02) and ≥moderate PVL (BE Myval 6.9% vs. SE ER 19.8%, OR 0.31, 95% CI 0.1–0.94; *p* = 0.0396) was significantly lower in the BE Myval group. Conclusions: In patients with severe symptomatic AS undergoing TAVR, the novel Myval BE THV provided a comparable performance to the well-known ER SE THV, and it was associated with a lower rate of PPI and ≥moderate PVL within 30 days and 6 months after the procedure. Randomized, head-to-head comparison trials are needed to confirm our results.

## 1. Introduction

During the last 20 years, transcatheter aortic valve replacement (TAVR) has progressively emerged as an effective alternative treatment option to surgical aortic valve replacement (SAVR) in a wide range of patients affected by symptomatic severe aortic valve stenosis [1,2,3,4,5]. Initially introduced among high-risk patients who were considered not suitable for SAVR, in the modern era, several randomized clinical trials (RCTs) have investigated the role of TAVR in subjects with intermediate- or even low-risk profiles, paving the way for a wider expansion of this less invasive procedure [2,3,5,6,7,8]. As the transcatheter approach expands in increasingly heterogenous populations, current evidence has addressed a head-to-head device comparison to determine significant differences in terms of clinical efficacy and early safety of the two major technical deployment systems available, either the balloon-expandable (BE) or the self-expanding (SE) transcatheter heart valves (THVs) [9,10,11,12]. The CHOICE trial was the first RCT comparing the two THVs, demonstrating a higher early device success with lower paravalvular leak (PVL) occurrence using the BE Sapien/XT THV (Edwards Lifesciences, Irvine, CA, USA) [9]. Since then, a few RCTs, including SOLVE-TAVI and SCOPE I trial, have addressed the differences with respect to the mechanism of deployment, size and device success, respectively, in the two technologies (even if with different THVs), showing excellent safety and efficacy profiles in multiple versions of the BE Sapien THV when compared with SE devices [2,9,11,12]. Recently, a novel BE THV (Myval, Meril Life Sciences Pvt. Ltd., Vapi, India) with features that ease procedural aspects, while theoretically maintaining favorable clinical outcomes as with the well-known Sapien THV series [13,14], entered the market. As very limited data are available regarding this novel THV, the purpose of our study was to compare the clinical performance of the BE Myval THV vs. the most commonly used SE THV Evolut R.

## 2. Materials and Methods

The EVAL (Evolut vs. Myval for the Treatment of Patients with Severe Aortic Valve Stenosis) is a single-center, retrospective cohort analysis including 166 consecutive patients with symptomatic, severe, native aortic valve stenosis who underwent TAVR from March 2019 to March 2021, either with the SE Evolut R THV or with the BE Myval THV at Istituto Clinico Sant’Ambrogio, Milan, Italy. The THV type and size were left to the discretion of the treating physician according to the patients’ clinical and anatomical characteristics. Severe aortic valve stenosis was defined according to the European Guidelines [15]. Multi-slice computed tomography (MSCT) was performed in all subjects to determine the anatomical characteristics of the aortic annular leaflet, and left ventricular outflow tract (LVOT) calcifications were classified and graded using a semiquantitative scoring system, as previously described [16].

Required pre-operatory evaluations, including medical history, electrocardiography, echocardiography, laboratory tests, assessment of coronary status and MSCT, along with the evaluation of risk scores and stratification among different risk profiles, were performed in all subjects before scheduling TAVR.

The study complied with the Declaration of Helsinki and was approved by local ethics committees. All patients provided written informed consent for the procedure and subsequent data collection based on local practice and/or local institutional review board approval. Local multidisciplinary heart teams evaluated all patients and confirmed the indications for TAVR. The transfemoral approach was preferred when feasible and the mode of anesthesia adopted was conscious sedation (CS) for almost all patients. Aortic valve pre-dilatation and THV post-dilatation were left to the operator’s discretion according to anatomical and clinical features. Periprocedural anticoagulation was performed with unfractioned heparin (UFH) as a standard of care and the release of the THV was angio-guided. Semiquantitative assessment of PVL was obtained during the procedure by assessing the relative density of contrast media in the left ventricle by cineangiography, and post-procedural evaluation of the hemodynamic transvalvular gradient was performed. In case of transfemoral procedures, the interventional access was pre-closed using a suture-based system with a dual Proglide (Abbott Vascular, Temecula, CA, USA), and a 0.018” wire (positioned via the contralateral access) was left in place during the procedure to favor an eventual balloon inflation after THV removal at the end of the procedure. In a minority of cases, Angioseal (St. Jude Medical, St. Paul, MN, USA) was used in addition to the double Proglide, while Prostar XL (Abbott Vascular, CA, USA) was used in case of Proglide failure.

After TAVR, long-term single anti-platelet therapy (e.g., aspirin 75–100 mg daily) was recommended, except in patients with an indication for oral anticoagulation or who had recently undergone percutaneous coronary intervention (PCI) with stent implantation (in this case, dual antiplatelet therapy was indicated). In “complex” situations (e.g., concomitant atrial fibrillation and PCI), the drug regimen and duration were decided on a case-by-case basis.

The primary endpoint of our study was early device success as defined by the third Valve Academic Research Consortium-3 (VARC-3) [17] consensus document, which is a composite endpoint including technical success (capturing peri-procedural technical success, safety and correct delivery of the device implanted), 30 days of freedom from all-cause mortality, surgery or intervention related to the device and early optimal performance of the valve (mean gradient < 20 mmHg, peak velocity < 3 m/s, Doppler velocity index ≥ 0.25, and less than moderate aortic regurgitation).

Secondary endpoints embodied a composite and singular components of 30 days of early safety, including all-cause mortality, cardiovascular mortality and (re)-hospitalization, peri-procedural neurologic events and the rate of disabling stroke measured using the modified Rankin scale (mRS), the occurrence of ≥moderate PVL, the incidence of peri- and post-procedural transient and permanent conduction disturbances, the rate of new permanent pacemaker implantation (PPI), major bleeding complications classified according to the VARC-3 new classification scheme, major access-related vascular and non-vascular complications, acute kidney injury (AKI) and peri-procedural myocardial infarction (MI), along with the New York Heart Association (NYHA) functional class and bioprosthesis function as assessed by transthoracic echocardiography (TTE).

Patients were re-evaluated at a 6-month follow-up and a composite endpoint of all-cause mortality and disabling stroke was considered. Secondary endpoints at the 6-month follow-up included ≥moderate residual PVL and PPI rate.

In particular, in the calculation of the rate of new PPI, patients with a prior permanent pacemaker who were not at risk for the outcome were excluded from the denominator.

The severity of PVL was assessed at baseline, at day 30 and at the 6-month follow-up, and graded using TTE according to established guidelines [15]. The assessment of THV stenosis included both flow-dependent (mean trans-prosthetic gradient ≥ 20 mmHg) and flow-independent parameters such as effective orifice area (EOA) and doppler velocity index (DVI). Severe patient–prosthesis mismatch (PPM) was defined as indexed effective orifice area (iEOA) ≤ 0.65 cm^2^/m^2^ in patients with a normal body mass index (BMI) and as iEOA ≤ 0.55 cm^2^/m^2^ in patients with a BMI > 30 kg/m^2^ [17].

Follow-up information was obtained by clinical visit or phone contact at day 30 and 6 months after the index procedure.

### Statistical Analysis

Data were presented as number and frequency for categorical and binary variables. Normality of variables was assessed by the Shapiro–Wilk test and normally continuous variables were described as mean ± standard deviation (SD). For non-normally distributed continuous variables, median with interquartile range (IQR) was used. Comparisons among the two groups (Myval vs. Evolut) were performed using a 2-sided, independent-samples Student’s t-test for normally distributed continuous variables and the Mann–Whitney–Wilcoxon test for non-normally distributed continuous data. Categorical variables were presented as proportions and compared by Fisher’s exact test or risk difference using the Cochran Mantel–Haenszel method. The differences in the rate of composite clinical endpoint for the Evolut R and the Myval groups were determined at the 95% confidence interval (CI). For all tests, a *p*-value < 0.05 (2-sided) was considered significant. Data were analyzed using SPSS version 26 (IBM, Armonk, NY, USA) and a general-purpose statistical software package called STATA (version: STATA 15.1, STATA Corp., College Station, TX, USA).

## 3. Results

**Figure 1:**
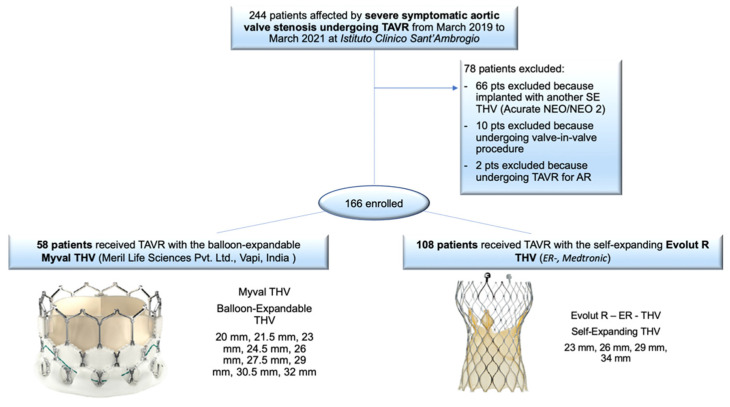
EVAL Registry flow-chart.

One hundred and sixty-six (166) patients with severe symptomatic aortic stenosis undergoing TAVR in a single center were retrospectively enrolled in a dedicated database between March 2019 and March 2021. One hundred and eight (108) patients received the SE Evolut R THV and fifty-eight (58) patients were treated with the BE Myval THV (Figure 1). All patients underwent follow-up until hospital discharge, and 30-day and 6-month clinical follow-up data were obtained for 98.8% of the patients (only 2 patients were lost at follow-up).

The two groups showed comparable baseline clinical characteristics (Table 1), with a mean age in the SE ER THV of 83 ± 5.7 vs. 82 ± 6 years in the Myval group (*p* = 0.66) and a slight, non-statistically significant, majority of male sex in the SE ER group (SE ER 61% vs. BE Myval 50%; *p* = 0.17). A higher prevalence of peripheral arterial disease in the BE Myval group was noted (SE ER 14% vs. BE Myval 31%; *p* = 0.01). Most patients were severely symptomatic, with a NYHA class ≥ III in 44.5% of the patients in the SE THV group vs. 50% of the subjects in the Myval arm. As for risk profiles, most patients exhibited a low- or intermediate-risk score, with very few subjects showing a high-risk score predicted with STS-PROM (STS-PROM ≥ 8% in 3.7% in the SE ER vs. 3.4% in the BE Myval; *p* = 0.92).

No significant difference was observed in terms of hemodynamic severity of the aortic valve stenosis in the two groups, with a mean aortic valve area of 0.75 ± 0.15 vs. 0.7 ± 0.18 cm^2^ (*p* = 0.25) and a mean aortic gradient of 41 ± 13 vs. 43.3 ± 13.8 mmHg (*p* = 0.23) in the SE ER and the BE Myval, respectively. Additionally, at the pre-procedural MSCT examination, a comparable annular diameter, amount of calcification and distance to coronary ostia were observed (Table 2).

The procedural characteristics in the two subgroups are summarized in Table 3.

Briefly, in most patients, a transfemoral approach was preferred, with only one subclavian approach in the SE Evolut R group and two in the BE Myval group. Almost all patients in the two groups received conscious sedation (CS) except for one patient who underwent subclavian access. Balloon pre-dilatation was performed in 54/58 patients in the BE Myval group (93%) and only in 31/108 patients (28.7%) in the SE Evolut R group (*p* < 0.001), while post-dilatation was performed less frequently in the BE Myval group (SE ER 27/108, 25% vs. BE Myval 2/58, 3.4%; *p* = 0.005) due to a lower incidence of ≥moderate PVL immediately after THV implantation. Cerebral protection devices were used in four patients (3.7%) in the SE ER group vs. three patients (5.2%) in the BE Myval group. The need for a second THV implantation occurred in only one case (0.9%) in the SE ER group (because of THV embolization), while conversion to cardiac surgery or SAVR did not occur in the analyzed cohort.

In-hospital outcomes in the two subgroups and detailed causes of device failure are listed in Table 4.

Intra-procedural death did not occur in any patient and the VARC-3 composite outcome of technical success upon exit from the procedure room was obtained in 105 patients (97.2%) in the SE ER group and in 57 patients (98.3%) in the BE Myval group (*p* > 0.9). Coronary artery obstruction occurred in three patients (2.8%) in the SE ER group and only in one patient (1.7%) in the BE Myval cohort (*p* = 0.66). The pre-discharge echocardiographic assessments of post-procedural PVL showed that the occurrence of any degree of PVL was more frequent in the SE group (SE-ER 40.8% vs. BE-Myval 20.7%; *p* = 0.01) and fewer patients in the BE Myval group experienced ≥moderate PVL after implantation (≥moderate PVL: BE Myval 3.4% vs. SE ER 14.8%; *p* = 0.0338).

**Figure 2:**
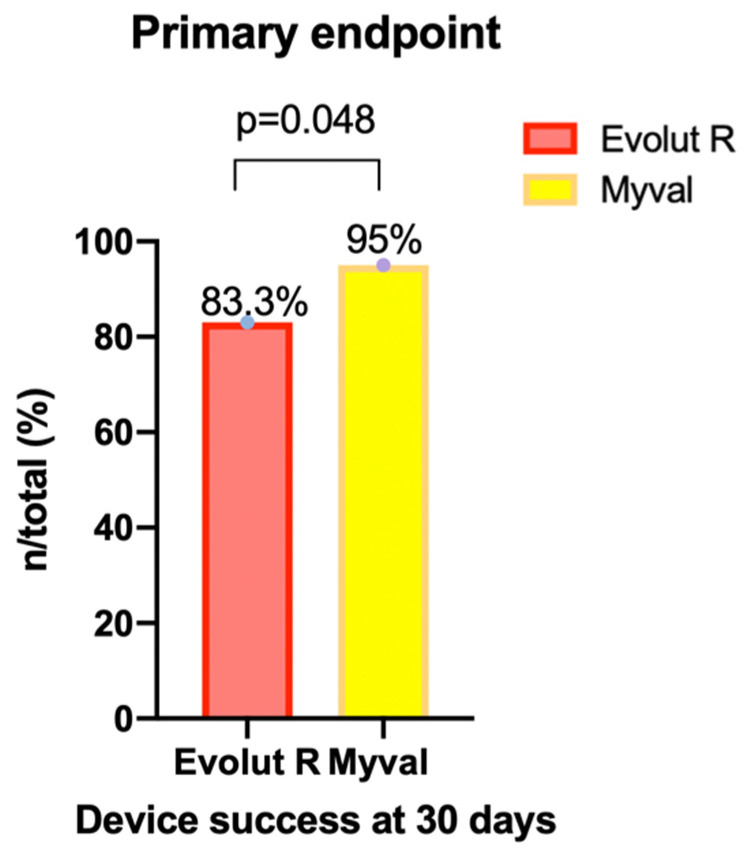
Primary composite endpoint occurrence in the balloon-expandable Myval THV and the self-expanding Evolut R THV groups.

Table 5 depicts the 30-day device success and early safety in the two subgroups in this study. In summary, at day 30, the primary composite endpoint of early device success, consisting of technical success, freedom from all-cause mortality, surgery or intervention related to the device and optimal device performance, was significantly higher in the BE Myval group, occurring in 55 patients (94.8%), compared to the SE Evolut R group, where it occurred in 90 patients (83.3%) (OR 3.667, 95% CI 1.094–12.14; *p* = 0.048) (Figure 2).

As for secondary endpoints and singular components of the primary endpoint, no significant differences were observed in the two cohorts when evaluating early (≤30 days from the index procedure) all-cause mortality (*p* > 0.9), cardiovascular mortality (*p* = 0.5), rate of disabling stroke (*p* = 0.35) and cardiovascular hospitalization (*p* = 0.16).

Other predefined secondary endpoints such as major vascular complications and VARC-3 ≥ type 2 bleeding complications were not different between the two cohorts (major vascular complications: BE Myval 1/58, 1.7% vs. SE ER 1/108, 0.9%, *p* > 0.9; ≥type 2 bleeding: BE Myval 3/58, 5.2% vs. SE ER 9/108, 8.3%, *p* = 0.54). Analogously, the incidence of AKI was comparable in the two subgroups, with one patient (1.7%) developing AKI in the BE Myval group and two patients (1.8%) in the SE ER group.

**Figure 3:**
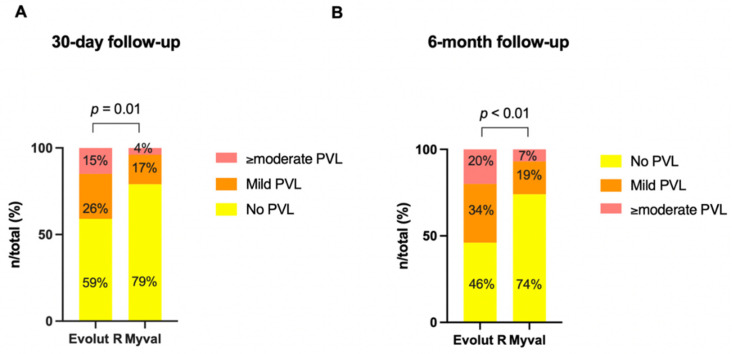
Incidence of none, mild or more than mild paravalvular leak between the balloon-expandable Myval vs. the self-expanding Evolut R THV at the 30-day follow-up (**A**) and the 6-month follow-up (**B**).

However, the proportion of patients who developed peri- and post-procedural conduction disturbances was significantly lower in the BE Myval group when compared to the SE THV cohort (BE Myval 27.6% vs. SE ER 44.4%, OR 0.48, 95% CI 0.24–0.96; *p* = 0.04), with a lower rate of PPI in the BE Myval group (BE Myval 11% vs. SE ER 24.2%, OR 0.38, 95% CI 0.15–0.99; *p* = 0.0535). Similarly, the proportion of patients with more than moderate PVL at day 30 was significantly lower in patients implanted with BE Myval THV (BE Myval 3.45% vs. SE ER 14.8%, OR 0.2, 95% CI 0.05–0.8; *p* = 0.0338) (Figure 3).

**Figure 4:**
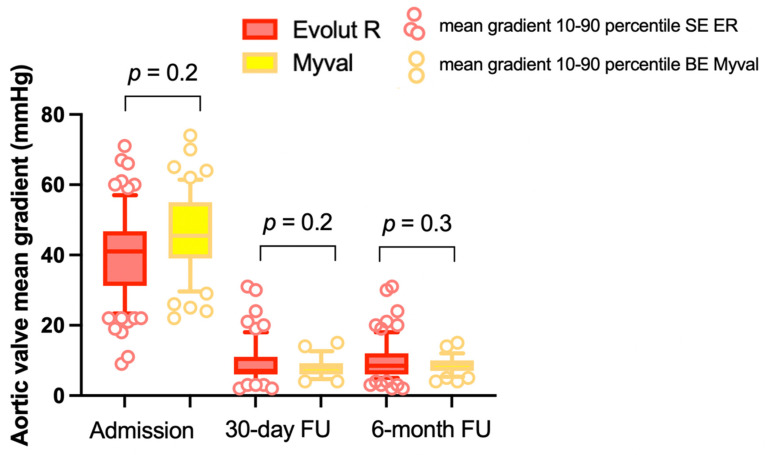
Mean aortic valve gradient at baseline, 30-day and 6-month follow-up in patients implanted with Evolut R THV and Myval THV, respectively.

Nonetheless, no significant difference was observed when evaluating the mean transvalvular gradient in the two groups (SE ER: 9.3 ± 4.9 mmHg vs. BE Myval: 8 ± 2.7 mmHg; *p* = 0.19) (Figure 4).

Consequently, the VARC-3-defined combined safety endpoint at day 30, consisting of freedom from all-cause mortality and surgery, all stroke, VARC-3 ≥ type 2 bleeding, major vascular complications, AKI type 3 and 4, ≥moderate PVL and new PPI, resulted to be significantly greater in the BE Myval cohort when compared to the SE ER group (BE Myval: 47/58, 81% vs. SE ER: 71/108, 66%, OR 2.227, 95% CI 1.038–4.902; *p* = 0.048).

As for functional capacity, symptomatic NYHA class improvement at day 30 occurred in most subjects, with 90% of the patients in the SE ER group and 89% of the patients in the BE Myval group being non-symptomatic or minimally symptomatic for dyspnea 30 days after the procedure (NYHA functional class ≤ II).

### Clinical Outcome at the Six-Month Follow-Up

At the 6-month follow-up, data regarding the composite endpoint were obtained for 98.8% of the study population and are summarized in Table 6.

Notably, the incidence of all-cause mortality and disabling stroke did not significantly differ in the two groups (BE Myval THV 6.9% vs. SE ER THV 15.1%, OR 0.42, 95% CI 0.15–1.2; *p* = 0.14). Contrarily, the proportion of PPI at 6 months was significantly lower in the BE Myval group, with only 6 patients (11%) implanting a permanent pacemaker compared to 25 patients (27.5%) in the SE group (OR 0.32, 95% CI 0.1273–0.8; *p* = 0.02).

**Figure 5:**
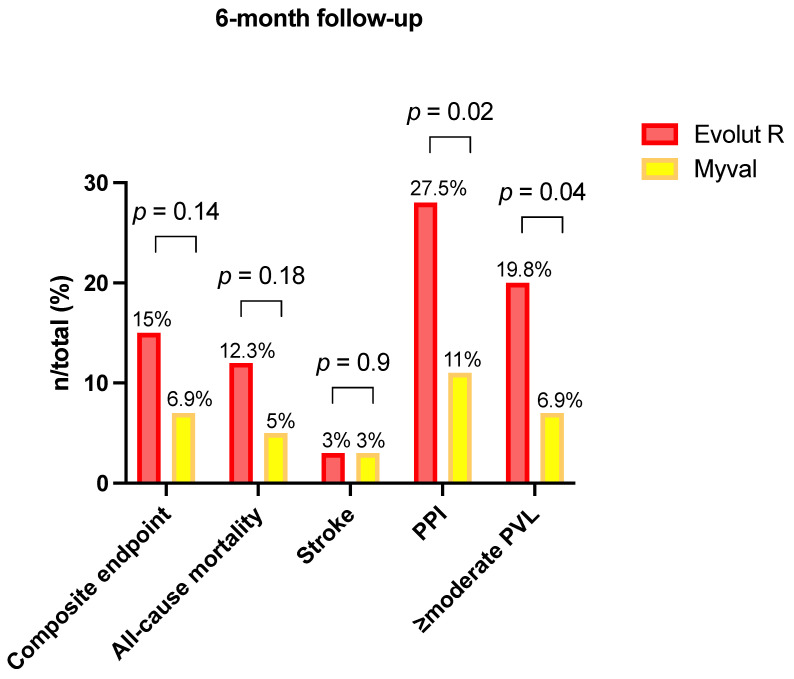
Secondary endpoints’ occurrence at the 6-month follow-up in patients treated with the balloon-expandable Myval vs. the self-expanding Evolut R.

Regarding 6-month echocardiographic THV performance, no differences were reported between the two groups in terms of mean aortic gradient (SE ER: 10 ± 5 mmHg vs. BE Myval: 9.2 ± 3 mmHg; *p* = 0.3). However, more than mild PVL appeared to be significantly lower in the BE Myval group (BE Myval 6.9% vs. SE ER 19.8%, OR 0.31, 95% CI 0.1–0.94; *p* = 0.0396), confirming what was observed in the pre-discharge and early assessment phase (Figure 5).

## 4. Discussion

To our knowledge, this is the first study analyzing clinical efficacy and early safety of the novel BE nickel cobalt frame Myval THV, compared to the well-known SE supra-annular ER bioprosthesis, in patients undergoing TAVR for severe, symptomatic, native aortic valve stenosis. Our findings demonstrated favorable clinical performance of the new BE Myval THV, with particularly low incidence of conduction system disturbances and hemodynamically significant PVL. Both the SE and the BE THVs provided significant functional improvement, along with comparable rates of overall mortality and disabling stroke in the peri-procedural setting and at the 6-month follow-up.

In the EVOLUT low-risk trial, TAVR with a SE supra-annular THV was noninferior to SAVR with respect to the risk of death or disabling stroke at 24 months and was associated with a lower proportion of disabling stroke, acute kidney injury and bleeding events [8]. However, a higher incidence of hemodynamically significant PVL and PPI was noted in the TAVR cohort. Notably, as equal or more than moderate PVL and conduction disturbances have been associated with increased mortality, major concerns have emerged in the performance of TAVR, especially in subjects with low-risk profiles and longer life expectancy [18,19,20,21].

The higher PVL rate with the SE THV was also confirmed by head-to-head comparisons with a cobalt chromium BE THV. In details, the CHOICE trial, comparing the BE THV Edwards Sapien XT with the SE THV Medtronic CoreValve, demonstrated a superior early device success in patients treated with a second-generation BE THV when compared to the SE counterpart [9]. In line with these results, the SOLVE-TAVI trial showed equivalent performance in terms of primary efficacy composite endpoints of all-cause mortality, stroke, moderate/severe PVL and PPI at day 30 of the newer generation SE and BE THVs, with a lower non-statistically significant rate of moderate/severe PVL in the BE THV cohort (SE THV 3.4% vs. BE THV 1.5%) while the PPI rate remained high with both THVs (23% vs. 19.2%, respectively, with the SE THV and the BE THV) [11].

In this setting, the newer generation BE Myval THV provided encouraging clinical results. In the single-arm Myval First trial, which was the first in-human prospective, multicenter, open-label study, evaluating 30 patients with a medium STS score of 6.4% ± 1.8%, this new-generation BE THV was associated with very low rates of peri-procedural and one-year overall mortality and minimal residual PVL and PPI rates (≥moderate PVL 0% and PPI rate 0%), supporting its use in intermediate- and high-risk patients with severe symptomatic native AS [13]. Such findings were confirmed by subsequent European multicenter studies that showed good hemodynamic performance and favorable clinical outcomes of the BE Myval THV, even in the context of low-risk patients. As a matter of fact, García-Gomez et al. demonstrated, in a cohort of 100 AS low-risk patients (medium STS score of 2.4% ± 0.8%), favorable short-term clinical outcomes and hemodynamic performance, along with an only 8% PPI rate [22]. Additionally, such profile of efficacy, with a particularly low rate of PPI and hemodynamically significant PVL, was also confirmed when compared with the most commonly used cobalt chromium BE THVs. Notably, in the first comparison of the Sapien-3 and the Myval THV in a case-matched population, the use of a nickel-cobalt frame was associated with favorable short-term early safety and clinical efficacy, along with a better transvalvular gradient, similar residual PVL and a lower PPI rate [22,23].

Our study, conducted in a cohort of intermediate–low-risk profile subjects, is the first comparison between the new BE Myval vs. the SE ER THV.

Among the 166 patients evaluated, we have shown an analogous clinical and hemodynamic performance between the two THVs, confirming a particularly favorable profile in terms of lower residual PVL and PPI rate with the BE Myval vs. the SE ER. The composite primary endpoint of early device success and the combined endpoint of early safety at day 30 were higher in the BE Myval cohort, which was attributed to a lower incidence of ≥moderate PVL and the less frequent need for PPI in patients implanted with the BE device.

These findings may be explained by the different technological design of the newly proposed Myval. The BE Myval is a novel BE THV consisting of bovine pericardium tissue, which receives an anti-calcification treatment known as AntiCa (Meril Life Sciences, Vapi, Gujarat, India) that forms a three-leaflet valve, showing both similarities (shape and intra-annular leaflets’ position) and differences when compared to the chromium-cobalt counterpart Sapien THV. In fact, such a device, which is equipped with a nickel-cobalt alloy support structure, displays a hybrid honeycomb cell design with a hexagonal frame comprising 53% of open cells on the upper half (to ensure un-jailing of the coronary ostia) and 47% of closed cells on the lower half (for high radial strength), as well as a relatively shorter frame height (17–20 mm). Such features may allow a more precise and favorable positioning with reduced ventricular depth of THV implantation. Following the positioning of the valve, it can be deployed across the native annulus, avoiding excessive throating between the left ventricular outflow tract, thereby reducing the risk of conduction disturbances and PPI. Furthermore, the presence of additional intermediate and extra-large sizes (e.g., 30.5 and 32 mm) of the BE Myval may play a key role in reducing the risk of PVL after implantation in extra-large annuli (actually not suitable for the treatment with SE THVs) [24].

Furthermore, in our study, the overall vascular complication rate was numerically higher in the SE ER group, with the vast majority related to the ancillary access and not to the interventional one (even though 43% of patients in the SE ER group required a 16F sheath access “pretreatment” to undergo sheatless TAVR with a 34 mm ER).

However, in the current analysis, the BE Myval was not compared with the latest development of the CoreValve family, the Evolut R Pro/Pro + THV, which is equipped with an external porcine pericardial wrap with the intent to reduce the proportion of PVL, while partially (a larger introducer sheath compared to its predecessor) maintaining the benefits of a low-profile SE THV. Thus, further direct THV comparisons are strongly needed to confirm our findings when the BE Myval THV is compared with the newest generation SE Evolut Pro/Pro + THV [25].

### Limitations

The main limitations of our study are the lack of randomization and the small sample size. As a matter of fact, our results, which may have significant practical implication in the near future for the use of the novel BE THV, need to be carefully interpreted, and randomized head-to-head comparison trials are needed to confirm these preliminary data. Indeed, the ongoing LANDMARK trial, which is a prospective, randomized, multinational study that will enroll approximately 768 patients undergoing TAVR for severe symptomatic aortic valve stenosis, allocated in a 1:1 randomization to Myval THV series or to contemporary THV, either Sapien THV or Evolut THV, may validate such preliminary evidence, elucidating the role and the potential benefit of the BE Myval THV for patients affected by severe symptomatic aortic stenosis undergoing TAVR [14]. Lastly, the short-term follow-up time and the single-center experience may potentially preclude from conclusive results on a long-term basis.

## 5. Conclusions

Our single-center, observational experience comparing a new BE THV with the SE ER THV demonstrated an equal clinical performance between the two THVs, while lower PVL and PPI rates were associated with the BE THV implantation. Larger and adequately powered randomized controlled trials are needed to confirm these initial findings.

## Figures and Tables

**Table 1:** Baseline clinical characteristics.

	Self-Expanding Valve (Evolut R) *n* = 108	Balloon-Expandable Valve (Myval) *n* = 58	*p*-Value
Age (years), mean ± SD	83 ± 5.7	82 ± 6	0.66
Male gender, *n* (%)	66/108 (61)	29/58 (50)	0.17
STS-PROM score (%), mean ± SD	3.9 ± 2.5	3.3 ± 1.8	0.07
PAD, *n* (%)	15/108 (14)	18/58 (31)	0.01 *
CAD, *n* (%)	52/108 (48)	35/58 (60)	0.1
Prior MI, *n* (%)	19/108 (18)	8/58 (14)	0.5
Prior PCI, *n* (%)	38/108 (35)	25/58 (43)	0.3
Prior CABG, *n* (%)	11/108 (10)	3/58 (5)	0.4
Atrial fibrillation, *n* (%)	41/108 (38)	18/58 (31)	0.1
Prior PM/ICD, *n* (%)	17/108 (16)	3/58 (5)	0.04 *
Prior stroke, *n* (%)	11/108 (10)	3/58 (5)	0.3
Renal insufficiency (GFR < 60 mL/min), *n* (%)	53/108 (49)	28/58 (48)	0.9
Pulmonary hypertension (PAPs > 60 mmHg), *n* (%)	7/108 (6.5)	4/58 (7)	0.9
COPD, *n* (%)	19/108 (18)	5/58 (9)	0.08
Cardiovascular risk factors			
- Diabetes mellitus, *n* (%)	33/108 (31)	12/58 (21)	0.1
- Arterial hypertension, *n* (%)	87/108 (81)	52/58 (90)	0.1
- Hypercholesterolemia, *n* (%)	54/108 (50)	35/58 (60)	0.2
- Former smoker, *n* (%)	29/108 (27)	18/58 (31)	0.7
- Obesity (BMI kg/m^2^), *n* (%)	18/108 (17)	4/58 (7)	0.07
New York Heart Association class, n (%)			
I	7/108 (6.5)	1/58 (1.7)	0.2
II	53/108 (49)	28/58 (48)	0.9
III	42/108 (39)	24/58 (41)	0.8
IV	6/108 (6)	5/58 (9)	0.4

STS score = Society of Thoracic Surgeons score; PAD = peripheral arterial disease; CAD = coronary artery disease; MI = myocardial infarction; PCI = percutaneous coronary intervention; CABG = coronary artery bypass graft; PM = pacemaker; ICD = implantable cardioverter defibrillator; GFR = glomerular filtration rate; COPD = chronic obstructive pulmonary disease; BMI: body mass index. * Statistically significant.

**Table 2:** Major echocardiographic and MSCT characteristics in the two groups.

	Self-Expanding Valve (Evolut R) *n* = 108	Balloon-Expandable Valve (Myval) *n* = 58	*p*-Value
**Transthoracic echocardiography**			
Aortic Valve Area (cm^2^), mean ± SD	0.75 ± 0.15	0.7 ± 0.18	0.2
Aortic Valve Area indexed (cm^2^/m^2^), mean ± SD	0.42 ± 0.1	0.41 ± 0.1	0.4
Aortic Valve mean gradient (mmHg), mean ± SD	41 ± 13	43.3 ± 13.8	0.2
LVEF (%), mean ± SD	52 ± 11	55.7 ± 10.2	0.07
Aortic regurgitation ≥moderate, *n* (%)	9/108 (8)	8/58 (14)	0.3
**Multislice Computed Tomography**			
Aortic annulus diameter (mm), mean ± SD	23.9 ± 3.1	23.8 ± 2.3	0.9
Aortic annulus area (mm^2^), mean ± SD	460.8 ± 105.7	466.9 ± 108.9	0.74
Aortic annulus average perimeter (mm), mean ± SD	77.3 ± 9.4	74.7 ± 7.4	0.3
Degree of aortic leaflets calcification (≥moderate), *n* (%)	56/108 (52)	32/58 (55)	0.7
Degree of annulus calcification (≥moderate), *n* (%)	16/108 (15)	9/58 (16)	0.9
Degree of LVOT calcification (≥moderate), *n* (%)	7/108 (6.5)	4/58 (7)	0.9
Height of coronary artery (mm), mean ± SD			
Left main	14 ± 3	13.7 ± 3.3	0.9
Right	15 ± 3.9	16.6 ± 3.5	0.2

LVEF = Left ventricular ejection fraction; LVOT = left ventricular outflow tract.

**Table 3:** Procedural characteristics.

	Self-Expanding Valve (Evolut R) n = 108	Balloon-Expandable Valve (Myval) n = 58	*p*-Value
Transfemoral TAVR, *n* (%)	107/108 (99)	56/58 (97)	0.27
Procedure time, min (mean ± SD)	113.2 ± 38	112.15 ± 38	0.6
Fluoroscopy time, min (mean ± SD)	32.2 ± 13.2	26.3 ± 9.8	0.005 *
Total contrast volume, mL	180 ± 61	179 ± 72	0.9
General anesthesia, *n* (%)	0/108 (0)	1/58 (1.7)	0.2
Pre-dilation, *n* (%)	31/108 (29)	54/58 (93)	<0.001 *
Cerebral protection device, *n* (%)	4/108 (3.7)	3/58 (5.2)	0.6
Implantation of multiple valves, *n* (%)	1/108 (0.9)	0/58 (0)	0.5
Need for SAVR, *n* (%)	0/108 (0)	0/58 (0)	>0.9
Post-dilatation, *n* (%)	27/108 (25)	2/58 (3.4)	0.005 *
Valve size (mm)			
21.5		5/58 (8.6)	
23	10/108 (9)	11/58 (19)	0.14
23.5			
24.5		13/58 (22.5)	
26	22/108 (20.3)	14/58 (24)	0.7
27.5		4/58 (7)	
29	30/108 (27.7)	7/58 (12.1)	0.03 *
30.5		2/58 (3.4)	
32		2/58 (3.4)	0.28
34	46/108 (43)		

SAVR = Surgical aortic valve replacement; TAVR = transcatheter aortic valve replacement; * statistically significant.

**Table 4:** In-hospital outcomes in the two subgroups.

	Self-Expanding Valve (Evolut R) *n* = 108	Balloon-Expandable Valve (Myval) *n* = 58	*p*-Value
Procedural death, *n* (%)	0/108 (0)	0/58 (0)	>0.9
Valve embolization, *n* (%)	1/108 (0.9)	0/58 (0)	0.5
Coronary artery obstruction, *n* (%)	3/108 (2.8)	1/58 (1.7)	0.6
Periprocedural MI, *n* (%)	2/108 (1.8)	1/58 (1.7)	0.9
Cardiac tamponade, *n* (%)	0/108 (0)	0/58 (0)	>0.9
Annular rupture, *n* (%)	0/108 (0)	0/58 (0)	>0.9
LV perforation, *n* (%)	0/108 (0)	0/58 (0)	>0.9
Ventricular arrhythmias, *n* (%)	3/108 (2.8)	1/58 (1.7)	0.6
Final PVL			
None/trace	64/108 (59.2)	46/58 (79.3)	0.01 *
Mild	28/108 (26)	10/58 (17.3)	0.2
≥Moderate	16/108 (14.8)	2/58 (3.4)	0.03 *
Technical success (VARC3), *n* (%)	105/108 (97.2)	57/58 (98.3)	>0.9

MI = Myocardial infarction; LV = left ventricle; PVL = paravalvular leak; VARC-3 = Valve Academic Research Consortium-3. * Statistically significant.

**Table 5:** Device success and early safety at day 30.

	Self-Expanding Valve (Evolut R) *n* = 108	Balloon-Expandable Valve (Myval) *n* = 58	*p*-Value
Device success (VARC 3)			
Primary endpoint, *n* (%)	90/108 (83.3)	55/58 (94.8)	0.048 *
Early safety (VARC 3), *n* (%)	71/108 (66)	47/58 (81)	0.048 *
All-cause mortality, *n* (%)	3/108 (2.8)	1/58 (1.7)	>0.9
Cardiovascular mortality, *n* (%)	2/108 (1.8)	0/58 (0)	0.5
Cardiovascular hospitalization, *n* (%)	5/108 (4.6)	0/58 (0)	0.2
Neurologic events, *n* (%)	2/108 (1.8)	2/58 (3.4)	0.6
Disabling stroke, *n* (%)	0/108 (0)	1/58 (1.7)	0.3
Non disabling stroke, *n* (%)	1/108 (0.9)	1/58 (1.7)	>0.9
Transient ischemic attack, *n* (%)	1/108 (0.9)	0/58 (0)	>0.9
Bleeding (VARC 3), *n* (%)	19/108 (17.6)	12/58 (20.7)	0.7
Type 1, *n* (%)	10/108 (9.3)	9/58 (15.5)	0.3
Type 2, *n* (%)	7/108 (6.5)	3/58 (5.2)	>0.9
Type 3, *n* (%)	1/108 (0.9)	0/58 (0)	>0.9
Type 4, *n* (%)	1/108 (0.9)	0/58(0)	>0.9
Vascular complications (VARC 3), *n* (%)	20/108 (18.5)	6/58 (10.3)	0.2
Major, *n* (%)	1/108 (0.9)	1/58 (1.7)	>0.9
Minor, *n* (%)	19/108 (17.6)	5/58 (8.6)	0.1
Access-related non-vascular complications, *n* (%)	1/108 (0.9)	0/58 (0)	>0.9
Conduction disturbances, *n* (%)	48/108 (44.4)	16/58 (27.6)	0.04 *
1st degree AV block, *n* (%)	6/108 (5.6)	1/58 (1.7)	0.42
2nd degree AV block, *n* (%)	4/108 (3.7)	2/58 (3.4)	>0.9
3rd degree AV block, *n* (%)	16/108 (14.8)	3/58 (5.2)	0.07
LBBB, *n* (%)	13/108 (12)	10/58 (17)	0.4
Ventricular arrhythmias, *n* (%)	3/108 (2.8)	0/58 (0)	0.5
Atrial fibrillation or flutter, *n* (%)	2/108 (1.8)	0/58 (0)	0.5
Permanent Pacemaker implantation, *n* (%)	22/91 (24.2)	6/55 (11)	0.053
Acute Kidney Injury, *n* (%)	2/108 (1.8)	1/58 (1.7)	>0.9
Prosthetic valve thrombosis, *n* (%)	0/108 (0)	0/58 (0)	>0.9
Prosthetic valve endocarditis, *n* (%)	0/108 (0)	0/58 (0)	>0.9
≥moderate paravalvular leak, *n* (%)	16/108 (14.8)	2/58 (3.4)	0.03 *
Aortic Valve mean gradient (mmHg), mean ± SD	9.3 ± 4.9	8 ± 2.7	0.2

VARC-3 = Valve Academic Research Consortium-3; AV block = atrioventricular block; LBBB = left bundle branch block. * Statistically significant.

**Table 6:** Clinical outcomes and echocardiographic characteristics at the 6-month follow-up.

	Self-Expanding Valve (Evolut R) *n* = 106/108	Balloon-Expandable Valve (Myval) *n* = 58/58	*p*-Value
Primary endpoint, *n* (%)	16/106 (15.1)	4/58 (6.9)	0.14
All-cause mortality, *n* (%)	13/106 (12.3)	3/58 (5.2)	0.18
Cardiovascular mortality, *n* (%)	10/106 (9.4)	2/58 (3.4)	0.22
Cardiovascular hospitalization, *n* (%)	8/106 (7.5)	3/55 (5.2)	0.75
Neurologic events, *n* (%)	3/106 (2.8)	2/58 (3.4)	>0.99
Disabling stroke, *n* (%)	1/106 (0)	1/58 (1.7)	>0.99
Non disabling stroke, *n* (%)	1/106 (0.9)	1/58 (1.7)	>0.99
Transient ischemic attack, *n* (%)	1/106 (0.9)	0/58 (0)	>0.99
Permanent Pacemaker implantation, *n* (%)	25/91 (27.5)	6/55 (11)	0.02 *
Prosthetic valve thrombosis, *n* (%)	0/106 (0)	0/58 (0)	>0.99
Prosthetic valve endocarditis, *n* (%)	0/106 (0)	1/58 (1.7)	>0.99
Paravalvular leak	57/106 (53.8)	15/58 (26)	<0.001 *
None/trace, *n* (%)	49/106 (46.2)	43/58 (74)	<0.001 *
Mild, *n* (%)	36/106 (34)	11/58 (19)	0.0138 *
≥moderate, *n* (%)	21/106 (19.8)	4/58 (7)	0.0396*
Aortic Valve mean gradient (mmHg), mean ± SD	10 ± 5	9.2 ± 3	0.3

* Statistically significant.

## References

[1] Cribier A., Eltchaninoff H., Bash A., Borenstein N., Tron C., Bauer F., Derumeaux G., Anselme F., Laborde F., Leon M.B. (2002). Percutaneous Transcatheter Implantation of an Aortic Valve Prosthesis for Calcific Aortic Stenosis. Circulation.

[2] Leon M.B., Smith C.R., Mack M., Miller D.C., Moses J.W., Svensson L.G., Tuzcu E.M., Webb J.G., Fontana G.P., Makkar R.R. (2010). Transcatheter Aortic-Valve Implantation for Aortic Stenosis in Patients Who Cannot Undergo Surgery. N. Engl. J. Med..

[3] Smith C.R., Leon M.B., Mack M.J., Craig D., Moses J.W., Svensson L.G., Tuzcu E.M., Webb J.G., Fontana G.P., Makkar R.R. (2011). Transcatheter versus Surgical Aortic-Valve Replacement in High-Risk Patients. N. Engl. J. Med..

[4] Adams D.H., Popma J.J., Reardon M.J., Yakubov S.J., Coselli J.S., Deeb G.M., Gleason T.G., Buchbinder M., Hermiller J., Kleiman N.S. (2014). Transcatheter Aortic-Valve Replacement with a Self-Expanding Prosthesis Abstract. N. Engl. J. Med..

[5] Leon M.B., Smith C.R., Mack M.J., Makkar R.R., Svensson L.G., Kodali S.K., Thourani V.H., Tuzcu E.M., Miller D.C., Herrmann H.C. (2016). Transcatheter or Surgical Aortic-Valve Replacement in Intermediate-Risk Patients. N. Engl. J. Med..

[6] Reardon M.J., Van Mieghem N.M., Popma J.J., Kleiman N.S., Søndergaard L., Mumtaz M., Adams D.H., Deeb G.M., Maini B., Gada H. (2017). Surgical or Transcatheter Aortic-Valve Replacement in Intermediate-Risk Patients. N. Engl. J. Med..

[7] Mack M.J., Leon M.B., Thourani V.H., Makkar R., Kodali S.K., Russo M., Kapadia S.R., Malaisrie S.C., Cohen D.J., Pibarot P. (2019). Transcatheter Aortic-Valve Replacement with a Balloon-Expandable Valve in Low-Risk Patients. N. Engl. J. Med..

[8] Popma J.J., Deeb G.M., Yakubov S.J., Mumtaz M., Gada H., O’Hair D., Bajwa T., Heiser J.C., Merhi W., Kleiman N.S. (2019). Transcatheter Aortic-Valve Replacement with a Self-Expanding Valve in Low-Risk Patients. N. Engl. J. Med..

[9] Abdel-Wahab M., Mehilli J., Frerker C., Neumann F.J., Kurz T., Tölg R., Zachow D., Guerra E., Massberg S., Schäfer U. (2014). Comparison of balloon-expandable vs. self-expandable valves in patients undergoing transcatheter aortic valve replacement: The CHOICE randomized clinical trial. JAMA—J. Am. Med. Assoc..

[10] Van Belle E., Vincent F., Labreuche J., Auffret V., Debry N., Lefèvre T., Eltchaninoff H., Manigold T., Gilard M., Verhoye J.P. (2020). Balloon-Expandable Versus Self-Expanding Transcatheter Aortic Valve Replacement: A Propensity-Matched Comparison from the FRANCE-TAVI Registry. Circulation.

[11] Thiele H., Kurz T., Feistritzer H.J., Stachel G., Hartung P., Eitel I., Marquetand C., Nef H., Doerr O., Lauten A. (2020). Comparison of newer generation self-expandable vs. balloon-expandable valves in transcatheter aortic valve implantation: The randomized SOLVE-TAVI trial. Eur. Heart J..

[12] Lanz J., Kim W.-K., Walther T., Burgdorf C., Möllmann H., Linke A., Redwood S., Thilo C., Hilker M., Joner M. (2019). Safety and efficacy of a self-expanding versus a balloon-expandable bioprosthesis for transcatheter aortic valve replacement in patients with symptomatic severe aortic stenosis: A randomised non-inferiority trial. Lancet.

[13] Sharma S., Rao R., Chandra P., Goel P., Bharadwaj P., Joseph G., Jose J., Mahajan A., Mehrotra S., Sengottovelu G. (2020). First-in-human evaluation of a novel balloon-expandable transcatheter heart valve in patients with severe symptomatic native aortic stenosis: The MyVal-1 study. EuroIntervention.

[14] Kawashima H., Soliman O., Wang R., Ono M., Hara H., Gao C., Zeller E., Thakkar A., Tamburino C., Bedogni F. (2021). Rationale and design of a randomized clinical trial comparing safety and efficacy of myval transcatheter heart valve versus contemporary transcatheter heart valves in patients with severe symptomatic aortic valve stenosis: The Landmark trial. Am. Heart J..

[15] Vahanian A., Beyersdorf F., Praz F., Milojevic M., Baldus S., Bauersachs J., Capodanno D., Conradi L., De Bonis M., De Paulis R. (2021). 2021 ESC/EACTS Guidelines for the management of valvular heart diseaseDeveloped by the Task Force for the management of valvular heart disease of the European Society of Cardiology (ESC) and the European Association for Cardio-Thoracic Surgery (EACTS). Eur. Heart J..

[16] Barbanti M., Yang T.H., Rodès Cabau J., Tamburino C., Wood D.A., Jilaihawi H., Blanke P., Makkar R.R., Latib A., Colombo A. (2013). Anatomical and procedural features associated with aortic root rupture during balloon-expandable transcatheter aortic valve replacement. Circulation.

[17] Généreux P., Piazza N., Alu M.C., Nazif T., Hahn R.T., Pibarot P., Bax J.J., Leipsic J.A., Blanke P., Blackstone E.H. (2021). Valve Academic Research Consortium 3: Updated endpoint definitions for aortic valve clinical research. Eur. Heart J..

[18] Sponga S., Perron J., Dagenais F., Mohammadi S., Baillot R., Doyle D., Nalli C., Voisine P. (2012). Impact of residual regurgitation after aortic valve replacement. Eur. J. Cardiothorac. Surg..

[19] Kodali S.K., Williams M.R., Smith C.R., Svensson L.G., Webb J.G., Makkar R.R., Fontana G.P., Dewey T.M., Thourani V.H., Pichard A.D. (2012). Two-Year Outcomes after Transcatheter or Surgical Aortic-Valve Replacement. N. Engl. J. Med..

[20] Herrmann H.C., Pibarot P., Hueter I., Gertz Z.M., Stewart W.J., Kapadia S., Tuzcu E.M., Babaliaros V., Thourani V., Szeto W.Y. (2013). Predictors of Mortality and Outcomes of Therapy in Low-Flow Severe Aortic Stenosis. Circulation.

[21] Karyofillis P., Kostopoulou A., Thomopoulou S., Habibi M., Livanis E., Karavolias G., Voudris V. (2018). Conduction abnormalities after transcatheter aortic valve implantation. J. Geriatr. Cardiol..

[22] García-Gómez M., Delgado-Arana J.R., Halim J., De Marco F., Trani C., Martin P., Won-Keun K., Montorfano M., den Heijer P., Bedogni F. (2021). Next-generation balloon-expandable Myval transcatheter heart valve in low-risk aortic stenosis patients. Catheter. Cardiovasc. Interv..

[23] Delgado-Arana J.R., Gordillo-Monge M.X., Halim J., De Marco F., Trani C., Martin P., Infusino F., Ancona M., Den Heijer P., Bedogni F. (2021). Early clinical and haemodynamic matched comparison of balloon-expandable valves. Heart.

[24] Kawashima H., Wang R., Mylotte D., Jagielak D., De Marco F., Ielasi A., Onuma Y., Den Heijer P., Terkelsen C.J., Wijns W. (2021). Quantitative Angiographic Assessment of Aortic Regurgitation after Transcatheter Aortic Valve Implantation among Three Balloon-Expandable Valves Quantitative Angiographic Assessment of Aortic Regurgitation after Transcatheter Aortic Valve Implantation among Three Balloon-Expandable Valves. Glob. Heart.

[25] Forrest J.K., Mangi A.A., Popma J.J., Khabbaz K., Reardon M.J., Kleiman N.S., Yakubov S.J., Watson D., Kodali S., George I. (2018). Early Outcomes With the Evolut PRO Repositionable Self-Expanding Transcatheter Aortic Valve with Pericardial Wrap. JACC Cardiovasc. Interv..

